# Sex-Specific Differences in the Placental Unfolded Protein Response in a Rodent Model of Gestational Hypoxia

**DOI:** 10.1007/s43032-022-01157-w

**Published:** 2022-12-27

**Authors:** Wen Tong, Esha Ganguly, Roberto Villalobos-Labra, Anita Quon, Floor Spaans, Dino A. Giussani, Sandra T. Davidge

**Affiliations:** 1 Department of Physiology, Development and Neuroscience, University of Cambridge, Cambridge, UK; 2 Centre for Trophoblast Research, University of Cambridge, Cambridge, UK; 3 Department of Obstetrics and Gynaecology, University of Alberta, Edmonton, Alberta Canada; 4 Department of Physiology, University of Alberta, Edmonton, Alberta Canada; 5 Women and Children’s Health Research Institute, University of Alberta, Edmonton, Alberta Canada

**Keywords:** Gestational hypoxia, Fetal growth restriction, Unfolded protein response, Sexual dimorphism

## Abstract

Gestational hypoxia is a major contributor to fetal growth restriction (FGR) and perinatal morbidity and mortality and has been closely linked to the activation of the unfolded protein response (UPR) in the placenta. Recent studies on adverse pregnancy conditions show differential adaptive responses in pregnancies carrying male or female fetuses. Here, we use an established rat model of hypoxic pregnancy and FGR to test the hypothesis that chronic hypoxia promotes sexually dimorphic activation of the placental UPR. Our data showed that gestational hypoxia increased glucose regulatory protein 78 (GRP78) expression in male placentae, increased activating transcription factor 6 activation (ATF6) in female placentae, and did not induce changes in other UPR markers. In addition, gestational hypoxia reduced fetal weight only in males and ATF6 activation correlated with an increase in the fetal crown-rump-length/body weight ratio only in females. These results suggest sex-specific divergence in the placental adaptive response to gestational hypoxia, which may account for the sexual dimorphism observed in placental function and pregnancy outcomes in complicated pregnancies.

**Graphical Abstract:**

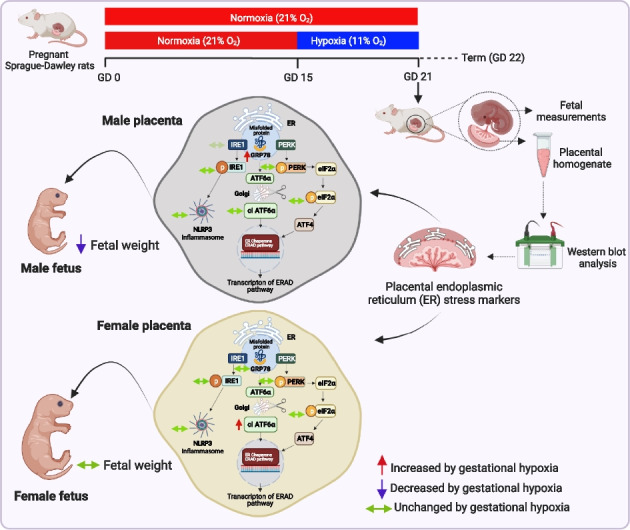

## Introduction

Chronic fetal hypoxia remains a serious obstetric complication and occurs in many conditions associated with placental compromise, including preeclampsia and FGR [1]. Placental hypoxia is closely linked to oxidative, mitochondrial, and endoplasmic reticulum (ER) stress [2, 3]. The ER responds to adverse conditions by activating pathways of the UPR including (1) the protein kinase RNA-like ER kinase (PERK) pathway, which inhibits non-essential protein synthesis through phosphorylation of eukaryotic initiation factor 2α (eIF2α); (2) the activating transcription factor 6 (ATF6) pathway, which increases protein folding capacity; and (3) the inositol-requiring enzyme 1α (IRE1α) pathway, which regulates cell survival [4]. Finally, uncompensated ER stress can trigger assembly of the NOD-, LRR-, and pyrin domain-containing protein 3 (NLRP3) inflammasome and apoptosis [4]. UPR activation has been reported in placentae from pregnancies complicated by high altitude, preeclampsia, and gestational hypoxia [5, 6]. However, whether gestational hypoxia affects placental UPR activation differentially in pregnancies carrying male and female fetuses in line with other studies of adverse pregnancy [7–9] is unknown. In this study, we tested the hypothesis that gestational hypoxia leading to FGR activates the placental UPR in a sexually dimorphic manner.

## Methods

### Animal Model

All procedures described are in accordance with guidelines of the Canadian Council on Animal Care (AUP #3693). The rat model of gestational hypoxia has been previously described [9]. In brief, on gestational day (GD) 15 (term: GD22), pregnant Sprague-Dawley dams were randomly assigned to either hypoxic (11% O_2_, GD15-21) or normoxic (21% O_2_) pregnancy. On GD21, dams were euthanized and whole placentae were isolated and immediately snap frozen in liquid nitrogen. Tissues were stored at −80°C until analysis.

### Molecular Analysis

The Western blotting protocol has been previously described [2]. In brief, 100 μg of protein per sample was separated on SDS-polyacrylamide gels by electrophoresis and transferred onto a nitrocellulose membrane. Membranes were treated with primary antibodies for GRP78, ATF6 and cleaved ATF6, eIF2α and phospho-eIF2α, IRE1α and phospho-IRE1α, and NLRP3. The next day, membranes were incubated with corresponding anti-rabbit or anti-mouse secondary antibodies. Blots were visualized with a LI-COR Odyssey Bioimager and quantified by densitometry compared to total protein staining. Total protein measurements were performed on the same samples used to detect the target protein.

### Statistical Analysis

Statistical analyses were performed using GraphPad Prism 9. All data are expressed as mean ± S.E.M. and analyzed using two-way ANOVA followed by Sidak’s post hoc tests: N male (●; *n*=7), H male (●; *n*=7), N female (**☐**; *n*=7), and H female (**☐**; *n*=7). *P*<0.05 was considered significant.

## Results

**Fig. 1:**
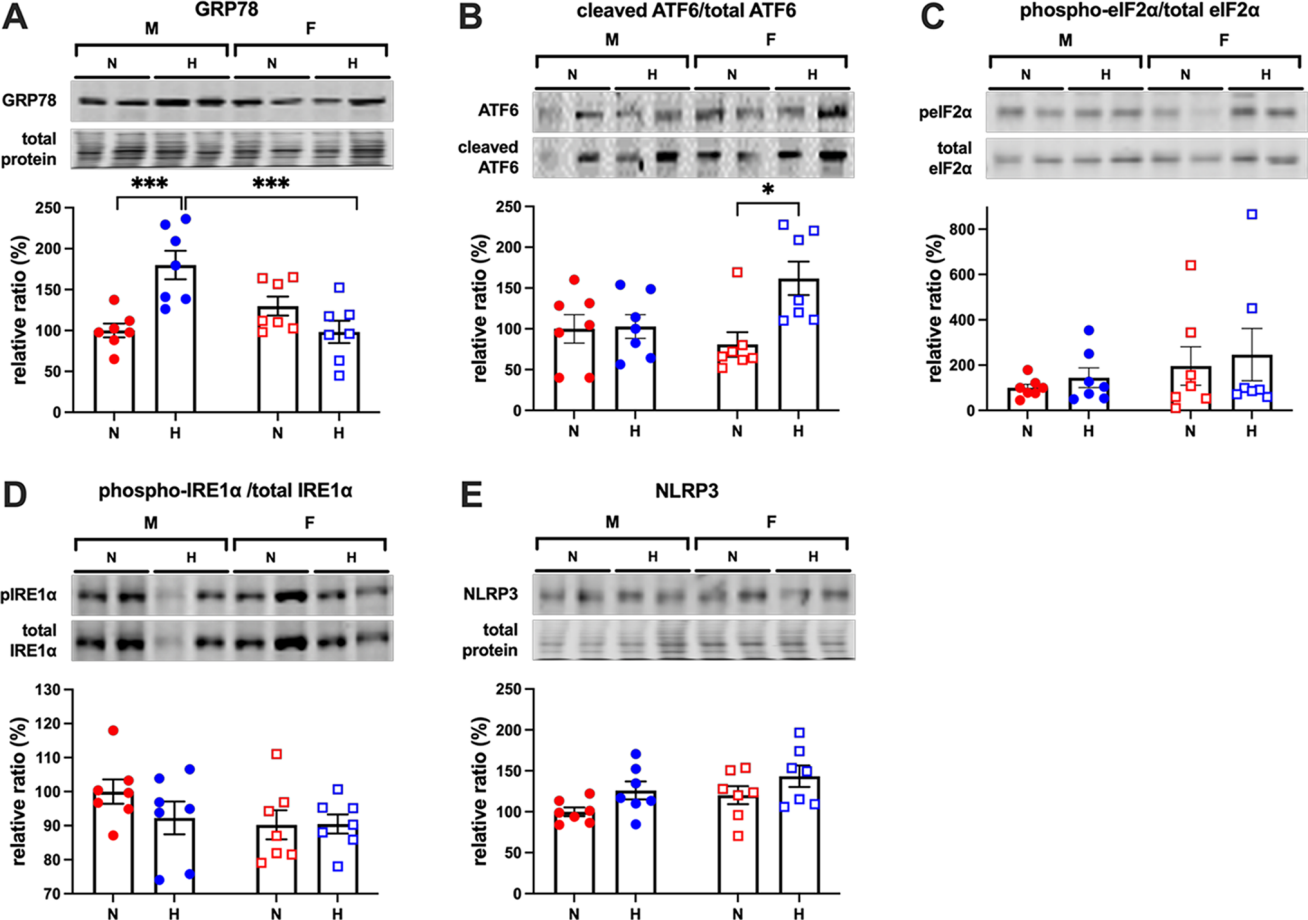
Values are mean ± S.E.M. for the relative ratio of GRP78/total protein (**A**), cleaved/total ATF6 (**B**), phosphorylated/total eIF2α (**C**), phosphorylated/total IRE1α (**D**), and NRLP3/total protein (**E**). Groups are male (M; ●) vs. female (F; **☐**) and normoxia (N; red) vs. hypoxia (H; blue). The representative images of the whole membrane for total protein staining in (**A**) and (**E**) were vertically compressed to allow the intensity of all bands within the lanes to be visualized. Significant differences are *P*<0.05, two-way ANOVA with Sidak’s post hoc test.

In placentae from male offspring, GRP78 protein levels were significantly increased by hypoxic compared to normoxic pregnancy and compared to placentae from female offspring subjected to gestational hypoxia (Fig. 1A). Levels of cleaved relative to total ATF6 were significantly increased in placentae from hypoxic female fetuses (Fig. 1B). Relative levels of the global eukaryotic initiation factor peIF2α (Fig. 1C), the UPR sensor phospho-IRE1α (Fig. 1D), and the inflammasome component NLRP3 (Fig. 1D) showed no differences between placentae from male and female fetuses or between placentae from normoxic or hypoxic offspring.

**Fig. 2:**
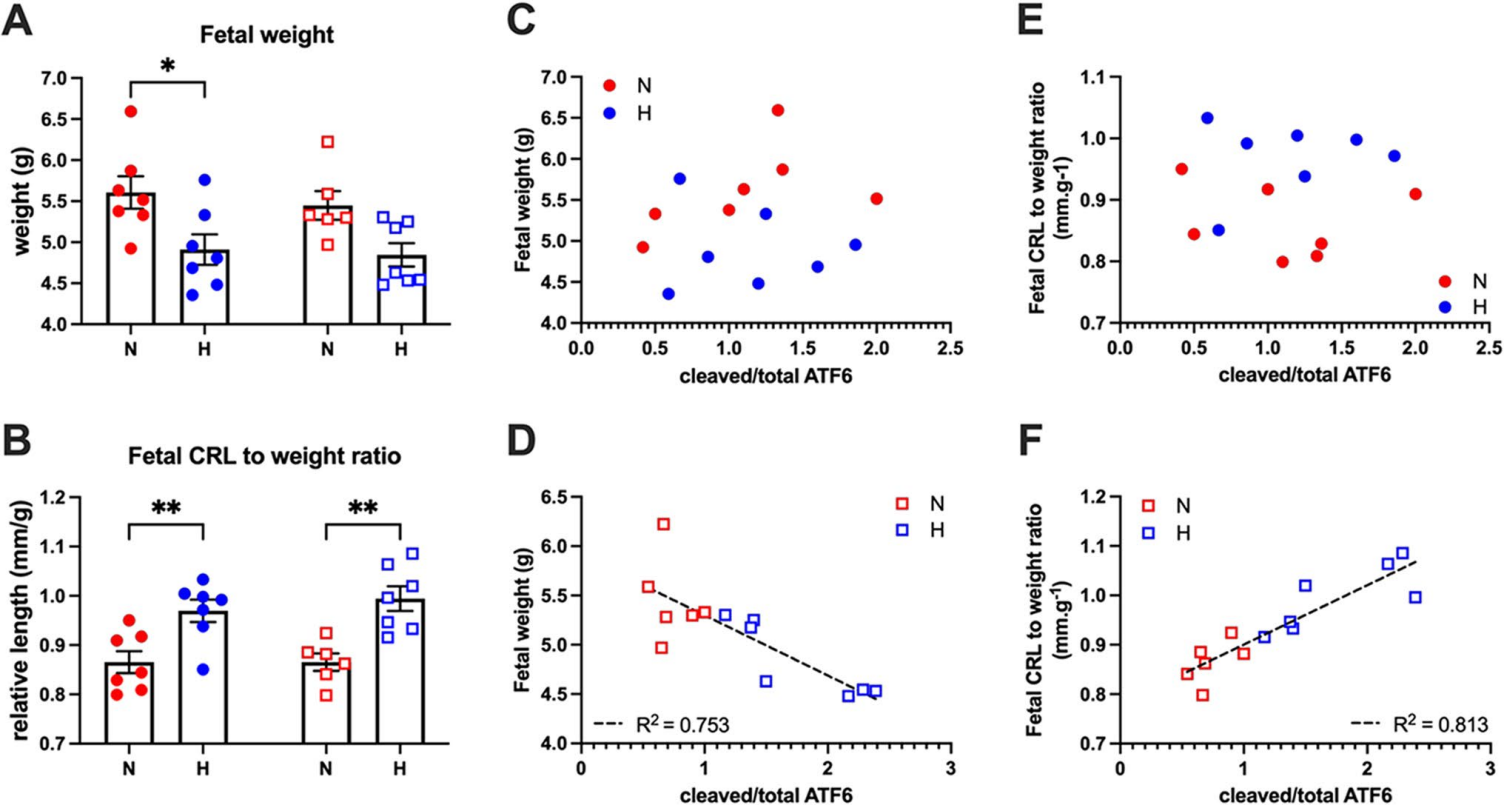
Values are mean ± S.E.M. for fetal weight (**A**) and fetal CRL/weight (**B**); the correlations of fetal weight and fetal CRL/weight with cleaved/total ATF6 in placentae from male (**C** and **E**) and female (**D** and **F**) fetuses. Groups are male (●) vs. female (**☐**) and normoxia (N; red) vs. hypoxia (H; blue). Significant differences are *P*<0.05, two-way ANOVA with Sidak’s post hoc test. Significant correlations (*P*<0.05) determined by Pearson’s correlation.

Gestational hypoxia significantly decreased fetal weight in male but not in female fetuses (Fig. 2A) [9]. The fetal crown-rump-length (CRL)/body weight ratio was increased in hypoxic compared to normoxic fetuses in both sexes (Fig. 2B). In female but not male fetuses, there was a significant correlation between placental levels of cleaved relative to total ATF6 with both fetal weight and fetal CRL/weight ratio (Fig. 2C–F).

## Discussion

In this study, we have shown sex-specific divergence in how the placental UPR increases fidelity of protein processing in response to hypoxic pregnancy, supporting the hypothesis tested. Male placentae show a rise in the chaperone protein GRP78 in response to hypoxia, which regulates ER homeostasis by guiding protein folding and assembly [4]. In contrast, female placentae show a relative increase in cleaved ATF6, suggesting activation of the endoplasmic reticulum-associated protein degradation pathway in female placentae only [4]. CRL/weight ratio is a measure of fetal leanness and asymmetric growth restriction, indicating significant changes to the growth hormone and insulin-like growth factor axis in response to hypoxia in both males and females [10]. However, ATF6 levels were inversely correlated with fetal weight and positively correlated with fetal CRL/weight ratio in females, but not in males, suggesting that ATF6-dependent protein degradation plays a less pertinent role for male intrauterine development. These sex-specific differences in the placental response to hypoxic pregnancy, with male placentae prioritizing protein stabilization while female placentae prioritize degradation of misfolded proteins, may contribute to sex differences in the fetal adaptive responses to a hypoxic environment. The data confirm that sex-specific differences should be considered when assessing alterations in the placental UPR.
